# Hydroxyapatite crystal deposition disease around the hip: a rare cause of piriformis syndrome and ischiofemoral impingement

**DOI:** 10.1259/bjrcr.20210075

**Published:** 2022-03-09

**Authors:** Elise Chua, Dhiren Shah

**Affiliations:** 1 Department of Radiology, London North West University Healthcare NHS Trust, London, United Kingdom

## Abstract

Hydroxyapatite crystal deposition disease (HADD) around the hip is typically described involving the gluteal tendons. However, HADD can occur in any location and result in varied clinical presentations. Even with small deposits, symptoms can be significant and imaging findings may appear aggressive, mimicking infection and malignancy particularly when in an atypical location.

We illustrate cases of both common and rare locations of HADD around the hip, in particular presenting as greater trochanteric pain syndrome, piriformis syndrome and ischiofemoral impingement. The latter two manifestations have not been previously described in the literature.

Low signal deposits were identified on MRI at the greater trochanter (gluteus medius tendon), proximal piriformis (adjacent to the sciatic nerve), and quadratus femoris (in the ischiofemoral space), respectively. Associated inflammatory changes with tendinopathy, bursitis and oedema were also demonstrated. The patient with piriformis syndrome underwent steroid injections and shockwave therapy with significant symptom improvement.

HADD should be within the differential diagnosis for hip pain and nerve compression syndromes. Knowledge of tendon anatomy and correlation with radiographs or CT, even after MRI, is crucial in recognising unusual manifestations and preventing unnecessary investigation. Therefore, we review the spectrum of imaging features of HADD, as well as the current evidence on its management, to confidently diagnose this condition.

## Introduction

Hydroxyapatite crystal deposition disease (HADD) is characterised by intra-articular or periarticular deposition of hydroxyapatite crystals. The most common manifestation is calcific tendinitis, which describes the deposition of hydroxyapatite crystals within tendons and occurs in up to 3% of adults, with peak incidence at 30 to 60 years of age.^1^ HADD is of uncertain aetiology, postulated to be a cell-mediated reactive process,^2,3^ distinct from degenerative tendinopathy.^4^

After the shoulder, the hip is the second most frequently involved site, seen in 5% of adults with calcific tendinitis.^5^ Although almost always described around the insertion of the gluteal tendons on the greater trochanter and/or gluteal tuberosity,^6^ HADD can occur in any tendon or muscle and hence have a variety of clinical manifestations.

Patients may be asymptomatic or present with: acute or chronic pain, tenderness, swelling, and restricted range of motion. The symptoms can be severe and are occasionally associated with low-grade fever and mildly raised inflammatory markers.^7^ Furthermore, imaging may demonstrate associated bony changes and soft tissue oedema.^7^ HADD can hence mimic conditions such as infection, tendon rupture, myositis ossificans and malignancy, and pose a diagnostic challenge particularly when in an atypical location.

Awareness of the anatomy of tendinous insertions and detecting calcific deposits on imaging is crucial to distinguish HADD from more aggressive pathology. We illustrate cases of both common and rare manifestations of HADD around the hip, in particular as piriformis syndrome and ischiofemoral impingement, which have not been previously described to our knowledge. We will review imaging features to facilitate a prompt diagnosis and the current evidence on management of HADD.

## Clinical presentations and imaging findings

### Case 1: Gluteus medius tendon – Greater trochanteric pain syndrome

**Figure 1.**
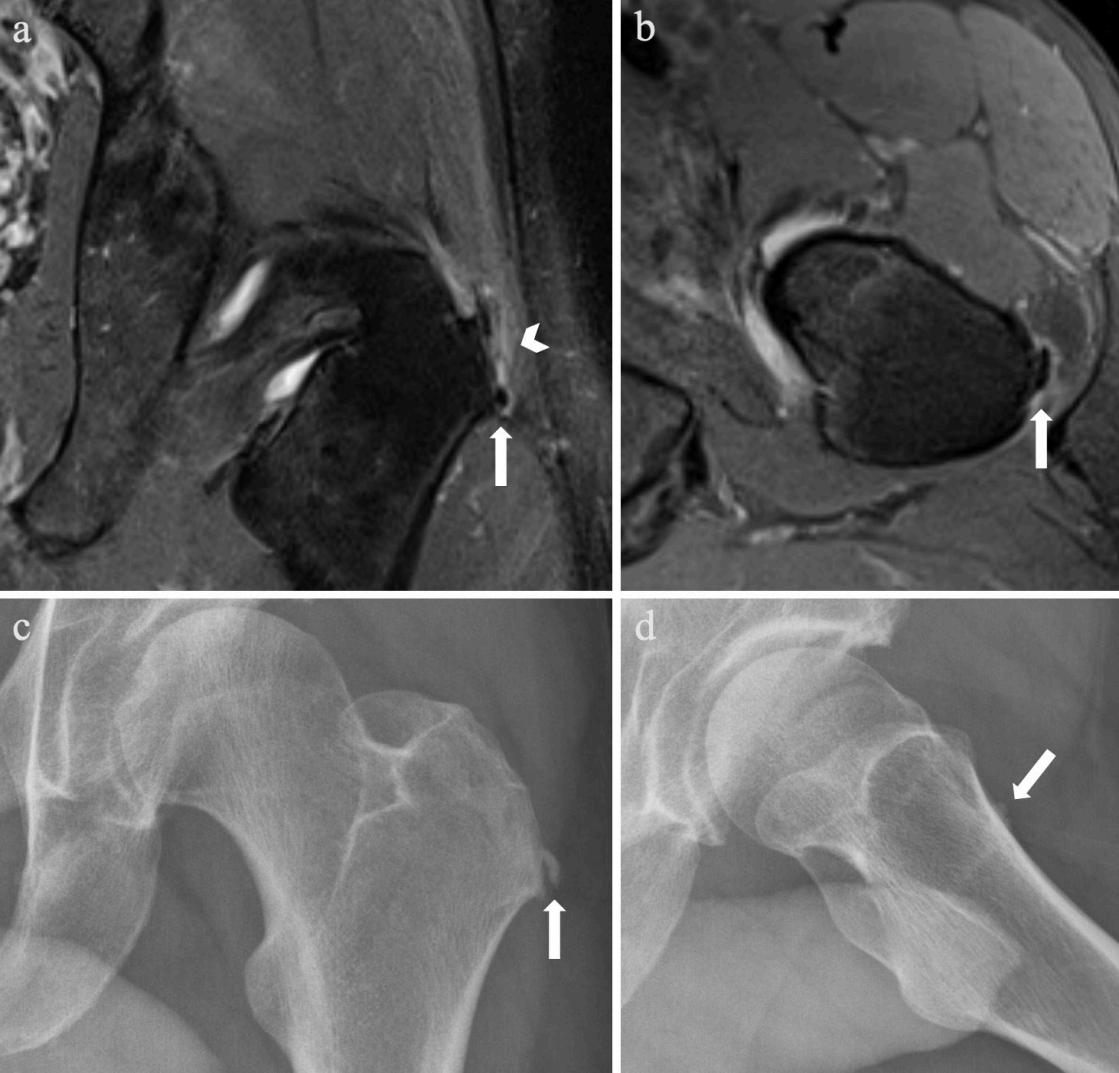
Left gluteus medius calcific tendinitis in a 46-year-old male presenting with greater trochanteric pain syndrome. Coronal (**a**) and axial (**b**) proton density (PD) fat-saturated (FS) MRI shows a 6-mm low signal focus at the inferior aspect of the left greater trochanter (lateral facet) (arrows), with increased signal in the adjacent left gluteus medius tendon and mild reactive trochanteric bursitis (arrowhead). Anteroposterior (**c**) and oblique (**d**) radiographs of the left hip show a corresponding calcific deposit at the left greater trochanter with slightly ill-defined edges (arrows).

A 46-year-old male presented with left lateral hip pain and stiffness, with focal tenderness over the greater trochanter. MRI demonstrated left gluteus medius insertional tendinopathy associated with a low signal focus at the left greater trochanter, confirmed to be a calcific deposit on a subsequent hip radiograph (Figure 1).

Greater trochanteric pain syndrome is characterised by pain and tenderness over the greater trochanter. It may be secondary to a diverse group of conditions, such as gluteus medius and/or minimus tendinopathy, trochanteric bursitis due to rheumatological disorders,^8^ and as in this case, gluteus medius calcific tendinitis. The pain can be anterior, superior or lateral depending on the facets of the greater trochanter affected.^7^

### Case 2: piriformis muscle – Piriformis syndrome

A 63-year-old female presented with a one-day history of acute severe left hip pain which radiated to her left calf, resulting in difficulty weight-bearing. The pain was worse on hip flexion and she was focally tender over the course of the sciatic nerve in the left gluteal region. Her symptoms were consistent with piriformis syndrome, which describes sciatic pain typically reproduced on internal rotation of a flexed hip. Other common features are: buttock pain; pain aggravated by prolonged sitting; tenderness near the greater sciatic notch; limitation of straight leg raise (positive Lasègue sign).^9^ Piriformis syndrome may be due to several entities, including an accessory piriformis muscle, muscle hypertrophy, trauma or mass lesion, which results in compression of the sciatic nerve at the greater sciatic notch.^10^

**Figure 2.**
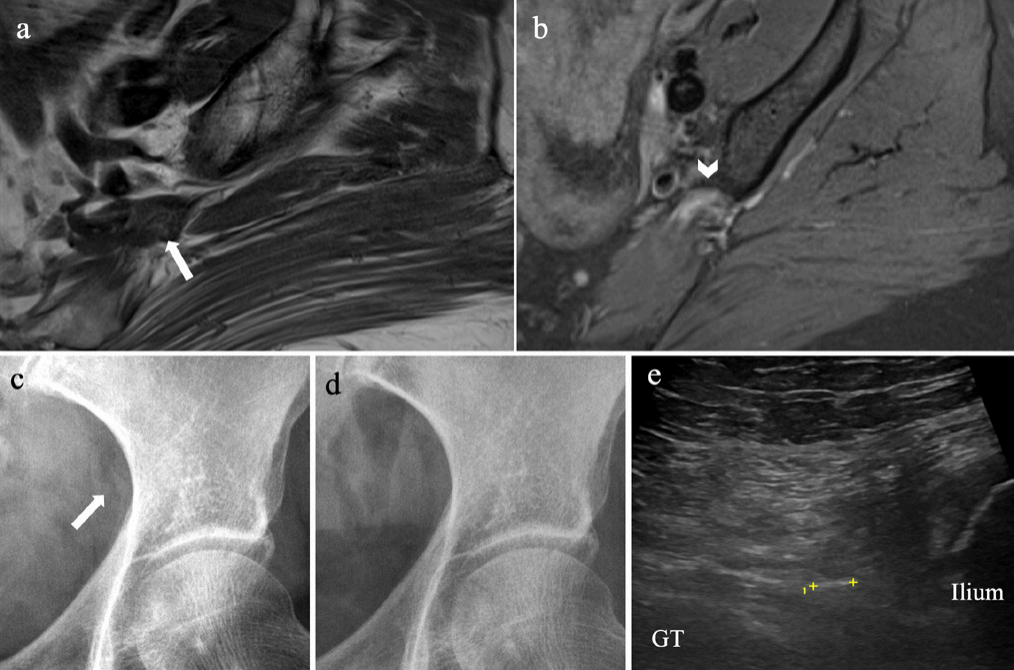
Piriformis HADD in a 63-year-old female with piriformis syndrome. Axial PD MRI (**a**) demonstrates a 9-mm low signal deposit within the proximal left piriformis muscle (arrow), closely applied to the sacral plexus converging into the sciatic nerve, with surrounding high signal (arrowhead) on axial FS sequences (**b**). Anteroposterior radiographs of the left hip show a faint 9 mm amorphous calcific density projected over the left greater sciatic notch, just medial to the ilium (arrow) (**c**). This resolved on the follow-up radiograph one-month later (**d**). On ultrasound, the calcific deposit was seen as an 8-mm hyperechoic focus within the left piriformis muscle (**e**), between the ilium and greater trochanter (GT) of the left femur.

A calcific deposit with surrounding oedema within the piriformis muscle was seen on MRI, closely applied to and likely resulting in irritation of the sciatic nerve due to inflammatory change (Figure 2a and b). This deposit was faintly visible on the initial radiograph (Figure 2c), in keeping with the resorptive phase of HADD. The calcific deposit was visible as a hyperechoic focus on ultrasound (Figure 2e) and direct pressure reproduced the patient’s symptoms of sciatica.

Due to the severity of her symptoms, she underwent a steroid injection around the deposits which provided significant immediate but short-term relief. She subsequently had low-energy shockwave therapy to this area with marked improvement in symptoms. The calcific deposit resolved on a follow-up radiograph one-month later (Figure 2d).

### Case 3: Quadratus femoris tendon

**Figure 3.**
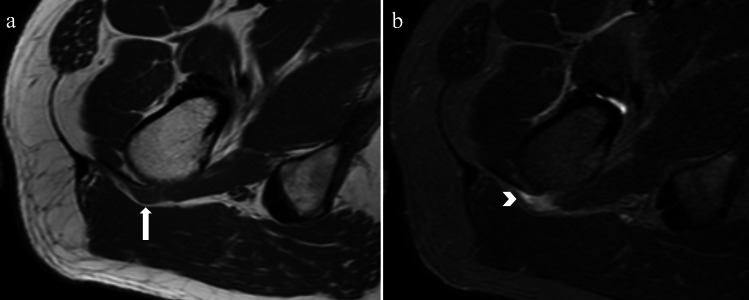
Right quadratus femoris calcific tendinitis in a 35-year-old male presenting with right hip pain. A 9-mm low signal deposit (arrow) is visualised at the right quadratus femoris insertion on axial *T*_1_-weighted MRI (**a**), with surrounding soft tissue oedema (arrowhead) on STIR sequences (**b**).

A 35-year-old male attended following a two-month history of right hip pain radiating to the lower back. On examination, he had reduced range of hip movement due to pain. MRI demonstrated a calcific deposit at the femoral insertion of the right quadratus femoris with surrounding oedema (Figure 3).

### Case 4: Quadratus femoris muscle – Ischiofemoral impingement

**Figure 4.**
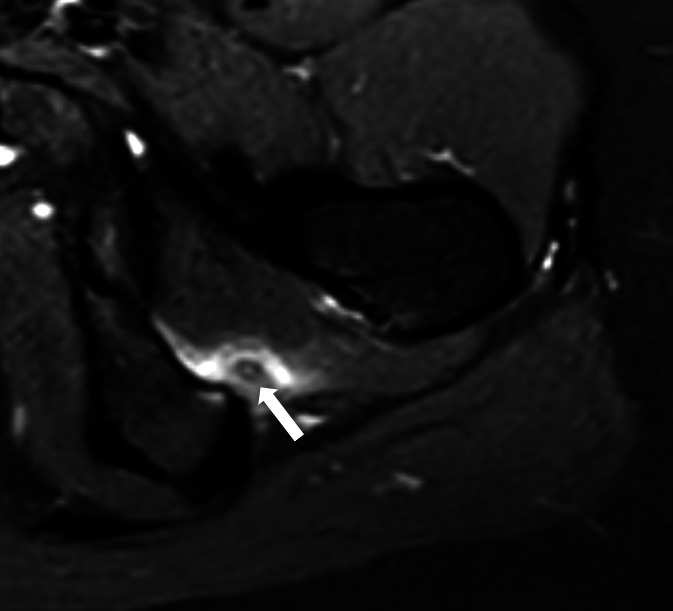
Left quadratus femoris HADD in a 25-year-old female presenting with ischiofemoral impingement. Axial PD FS MRI shows a 6-mm low signal deposit within the left quadratus femoris muscle (arrow), associated with marked high signal and expansion in keeping with muscle oedema.

Lastly, a 25-year-old female presented with three months of left hip pain on weight bearing, sitting on hard surfaces and stretching her left leg. On examination, symptoms were worse on extension and external rotation. MRI demonstrated a low signal deposit within the left quadratus femoris muscle, just lateral to the left ischial tuberosity, narrowing the ischiofemoral space and associated with considerable surrounding muscle oedema (Figure 4). Findings were in keeping with ischiofemoral impingement syndrome, an increasingly recognised cause of hip pain due to impingement of the quadratus femoris muscle between the ischial tuberosity and lesser trochanter.

Symptoms can be non-specific but typically reproduced on hip extension, adduction and external rotation.^11^ Other causes include anatomic variants of the ischium or femur, developmental hip dysplasia, ischial tuberosity enthesopathies, and mass lesions.^12^

Although it was not possible to obtain histopathological correlation as none of the cases proceeded to needle aspiration or surgery, the imaging features described are consistent with that seen in HADD. None of the patients had a relevant history of trauma or infective symptoms. They had normal inflammatory markers, as well as normal biochemical and bone profiles. In cases 1, 3 and 4, the patients’ symptoms resolved with conservative management indicating a benign process.

## Discussion

Although the pathogenesis of HADD remains uncertain, several theories have been proposed. Bishop^13^ and Bosworth^1^ postulated a degenerative process, whereby ischaemia or repetitive trauma results in hyaline degeneration and calcium deposition. This theory has, however, been contradicted by subsequent observations, namely, the relatively early peak incidence of HADD,^1^ complete resolution of HADD in certain cases^4^ and different composition of calcific deposits compared to degenerative tendinopathy.^14^ Benjamin hypothesised a process similar to endochondral ossification of fibrocartilage at tendon insertions.^15^

The most well-described pathogenesis is by Uhthoff et al, who proposed a reactive or cell-mediated process with progressive stages which have distinct imaging features and often correlate with clinical symptoms.^3^ In the formative phase, impaired perfusion due to vascular or mechanical factors result in local hypoxia, triggering fibrocartilaginous metaplasia and deposition of calcium hydroxyapatite within extracellular matrix vesicles.^16^ This is followed by a resting phase for a variable period of time. During these initial stages, the calcific deposit is typically homogeneous and round/ovoid in shape. Patients tend to be asymptomatic or have chronic pain secondary to mechanical impingement.

During the resorptive phase, the deposit undergoes phagocytosis by macrophages and multinuclear giant cells.^17^ This results in oedema and increased intratendinous pressure. The deposit resembles ‘toothpaste’ in consistency and can thus rupture into nearby tissues, including the musculotendinous junction and muscle, and induce an inflammatory reaction. The calcification appears ill-defined, heterogenous and fluffy, and often correlates with acute clinical symptoms.^18^ Finally in the reparative phase, fibroblasts incite new collagen fibre formation and restore the tendon collagen pattern.^19^

A more recent theory is that HADD is caused by the erroneous differentiation of tendon-derived stem cells into calcium-depositing chondrocytes or osteoblasts instead of tenocytes, due to altered local conditions such as excessive mechanical loading and microinjuries.^20,21^

Increased incidence of HADD has been observed in patients with diabetes,^22^ thyroid and oestrogen endocrine disorders,^23^ certain genes such as the HLA-A1 genotype,^24^ and with variations in tissue transglutaminase two and osteopontin.^25^ However, the exact correlation and underlying pathogenesis is still unclear.

Calcifications may range in size from a few millimetres to centimetres, but no relationship between the size and severity of pain has been shown.^26^ Radiographs are helpful and cost-effective in characterising the contour and extent of calcific deposits and thus inference of the phase of the disease.

CT is sensitive for the detection of hydroxyapatite deposits and a comet-tail appearance is characteristic.^27^ If there is a confounding history of trauma, HADD may be mistaken for avulsion fragments in the acute setting, or heterotopic ossification/myositis ossificans in chronic HADD. CT is particularly useful in this context as it can evaluate the morphology and consistency of deposits. This enables differentiation from ossification or avulsion fragments, which have corticated margins and are of higher density (100–400 HU for hydroxyapatite versus 700–1500 HU for bone).^28^ CT is hence also important if planning intervention, such as needle aspiration.

Whilst ultrasound is less useful for the diagnosis of HADD, it is commonly used to guide treatment. A range of sonographic appearances have been described; hyperechoic arc-shaped foci with posterior acoustic shadowing is suggestive of the formative and resting phases, whilst deposits can appear nodular, fragmented, or cystic, with or without acoustic shadowing, during the resorptive phase.^29^ Increased vascularity on power Doppler may be seen in the acute phase, although in only a third of cases.^30^

Calcific deposits appear as focal areas of low signal on all MRI sequences, typically near tendon insertions. Whilst MRI is the best modality for assessment of inflammatory changes and for other causes of hip pain, it is important to appreciate HADD can have an aggressive appearance. During the acute resorptive phase, associated oedema can be extensive and mimic trauma or infection.^7^ Cortical erosion, periosteal reaction and marrow involvement have been described and may be confused for an infective or neoplastic process such as a chondroid lesion.^27^

Detection of characteristic calcification near or within a tendon, in conjunction with the absence of a soft-tissue mass, is thus crucial for diagnosis. Small deposits may be difficult to visualise on MRI and hence if HADD is suspected, particularly in an uncommon location, radiographs can be obtained to confirm the diagnosis. Gradient echo pulse sequences could also be added to the protocol to utilise the susceptibility artefact to highlight calcification.

Painful HADD is typically managed with nonsteroidal anti-inflammatory drugs and physical therapy. HADD is usually a self-limiting process, with studies showing clinically significant improvement in 72% of patients with calcific tendinitis of the shoulder^26^ and 80% of patients with calcific tendinitis of the hip^31^ with conservative treatment. If symptoms persist and/or significantly impact quality of life, steroid injection, ultrasound or CT-guided needle aspiration and lavage (*i.e.,* barbotage)^32^ and extracorporeal shockwave lithotripsy^33^ have shown to provide symptomatic relief and reduction in the size of deposits. However, there is little consensus on which minimally invasive therapy is more effective, and with the majority of trials evaluating calcific rotator cuff tendinitis. Meta-analyses showed only weak evidence that ultrasound-guided lavage is more effective than a subacromial corticosteroid injection,^34,35^ although needling combined with a corticosteroid injection has been shown to be more effective than injection alone^36^ or extracorporeal shockwave lithotripsy.^37–39^ In refractory cases, surgical resection may be considered.^31^

HADD can occur in unusual locations, present with acute and severe symptoms, and imaging appearances can be aggressive with bony erosion and extensive soft tissue or marrow oedema. HADD can thus pose a diagnostic challenge, mimicking trauma, infection or malignancy. Radiographs can be especially helpful, even after initial imaging with MRI to visualise small deposits. Although an uncommon entity, HADD should be considered in the differential diagnosis of acute or chronic hip pain and nerve compression syndromes. Knowledge of tendon anatomy and the range of imaging manifestations of HADD is critical in recognising HADD when it presents in atypical locations, to promptly diagnose the condition, and prevent unnecessary workup or intervention. Further studies comparing the effectiveness of different minimally invasive therapies, particularly for HADD around the hip, is needed.

## Learning points

- Clinicians and radiologists should be alert to atypical manifestations of hydroxyapatite crystal deposition disease, which can present with acute pain and nerve compression syndromes.
- Imaging findings can appear aggressive and mimic trauma, infection, or malignancy.
